# Lesion Preparation Before Coronary Intravascular Brachytherapy: A Comparison of Plain Balloon Versus Cutting/Scoring Balloon Angioplasty

**DOI:** 10.1016/j.jscai.2025.103858

**Published:** 2025-08-19

**Authors:** Gal Sella, Chloe Kharsa, Mangesh Kritya, Devin Olek, Bin S. Teh, Muhammad Faraz Anwaar, Joseph Elias, Elia El Hajj, Albert E. Raizner, Andrew Farach, Neal S. Kleiman, Alpesh Shah

**Affiliations:** a The Heart Center, Kaplan Medical Center, Rehovot, Israel; b Department of Cardiology, Houston Methodist DeBakey Heart and Vascular Center, Houston, Texas; c Department of Radiation Oncology, Houston Methodist Hospital, Houston, Texas

**Keywords:** balloon angioplasty, cutting balloon, in-stent restenosis, intravascular brachytherapy, scoring balloon, target lesion revascularization

## Abstract

**Background:**

Coronary intravascular brachytherapy (IVBT) has remained as an effective treatment for recurrent in-stent restenosis (ISR). However, optimal lesion preparation techniques prior to radiation delivery remain undefined. This study evaluated the clinical outcomes of IVBT after lesion preparation with either plain balloon angioplasty or cutting/scoring balloon angioplasty.

**Methods:**

We conducted a retrospective analysis of 219 patients who underwent vascular brachytherapy for coronary ISR between June 2016 and January 2024 at the Houston Methodist Hospital. Patients were stratified based on the type of balloon used for lesion preparation: plain balloon (n = 140) or cutting/scoring balloon (n = 79). The primary end point was target lesion revascularization (TLR) at 1 year. Secondary end points included major adverse cardiovascular events, stent thrombosis, and bleeding complications.

**Results:**

Baseline demographic characteristics were similar between groups, except for older age in the cutting/scoring balloon group (67.0 ± 11.0 vs 64.0 ± 10.3 years; *P* = .047). Lesion length was comparable (28.01 ± 18.62 vs 26.49 ± 17.16 mm; *P* = .55). At 1-year follow-up, TLR rates were similar (26.6% vs 17.9%; *P* = .17), as were major adverse cardiovascular event rates (32.9% vs 35.0%; *P* = .86).

**Conclusions:**

We observed no significant difference in 1-year and 3-year clinical outcomes compared to conventional plain balloon angioplasty. The trend toward higher TLR rates in the cutting/scoring balloon group warrants further investigation in larger, prospective studies. These findings suggest that lesion-specific factors, rather than balloon type alone, may be more important determinants of outcomes after IVBT for ISR.

## Introduction

In-stent restenosis (ISR) remains a significant challenge in interventional cardiology, despite advances in-stent technology and antiproliferative drug elution. When conventional treatments fail, intravascular brachytherapy (IVBT) is considered an effective modality for recalcitrant ISR.^1^ This approach delivers localized radiation to inhibit neointimal hyperplasia through DNA damage to rapidly dividing cells, disrupting the biological cascade that leads to restenosis.^2^

Successful IVBT requires proper lesion preparation to ensure optimal lumen gain and radiation delivery.^3^ Traditionally, this preparation has involved dilating the stenotic segment with a conventional balloon catheter through radial force application. However, this approach may be limited by elastic recoil and nonuniform plaque modification, potentially compromising the efficacy of subsequent radiation therapy.

Specialized balloon technologies, such as cutting and scoring balloons, have been developed to address these limitations. Cutting balloons incorporate microsurgical blades (atherotomes) that create controlled, longitudinal incisions in the plaque and underlying vessel wall during balloon inflation. Similarly, scoring balloons feature wire or nitinol elements that score the plaque during inflation. These mechanical modifications theoretically allow more controlled plaque fracture, reduced vessel trauma, decreased elastic recoil, and enhanced lumen gain than conventional balloon angioplasty.^4^ Additionally, increased friction between balloon and vessel limits balloon slippage.^5^

The potential advantages of cutting/scoring balloons in lesion preparation before IVBT include more uniform plaque modification, potentially allowing more homogeneous radiation delivery; reduced barotrauma and subsequent inflammatory response; and decreased incidence of dissections requiring additional stenting, which could interfere with radiation delivery and potentially lower risk of geographic miss due to more precise lesion treatment.^4^

Despite these theoretical advantages, there is limited contemporary evidence comparing the effectiveness of different balloon types for lesion preparation before IVBT.^5^, ^6^, ^7^ This knowledge gap is particularly relevant given the resurgence of IVBT as a treatment option for recalcitrant ISR. Understanding the impact of lesion preparation strategy on IVBT outcomes could help optimize treatment protocols and improve patient care.

In this article, we present a comparative analysis of 1-year and 3-year outcomes after IVBT for ISR with lesion preparation using either conventional plain balloon angioplasty or cutting/scoring balloon angioplasty. By examining the differences in procedural success, complications, and long-term outcomes between these approaches, we aim to provide insights into the optimal lesion preparation strategy before IVBT.

## Materials and methods

### Study population and data collection

We conducted a retrospective analysis using data from the Houston Methodist Hospital Brachytherapy Registry, which is one of the largest single-center collections of vascular brachytherapy procedures. The registry includes patients who underwent vascular brachytherapy for ISR between June 2016 and January 2024. For this analysis, we included 219 patients who had plain or cutting balloon for lesion preparation; 12 of them (5.5%) were lost to follow-up at 1 year. Lesions that required lithotripsy or laser atherectomy were excluded from the study. All procedures were performed at our tertiary care center using standardized protocols.

Data collection utilized established registry protocols with comprehensive databases capturing detailed patient information, including baseline demographic characteristics, cardiovascular risk factors, clinical presentation, previous cardiac history, and complete procedural characteristics. Postprocedural follow-up employed comprehensive medical record review supplemented by clinical encounters. Repeat coronary angiography was performed when clinically indicated, such as in cases of recurrent symptoms or objective evidence of ischemia. The follow-up data collection process included documentation of all major adverse cardiac events, with particular attention to target lesion revascularization (TLR) procedures, myocardial infarctions, and cardiovascular-related hospitalizations. If data were missing, phone calls were permitted to obtain the missing information.

### Patient classification

Patients were stratified into 2 groups based on the type of balloon used for lesion preparation before brachytherapy: those treated with conventional plain balloon angioplasty (plain balloon group, n = 140) and those treated with cutting or scoring balloon angioplasty (collectively referred as cutting balloon group in this article, n = 79). The choice of balloon type was left to the discretion of the operating interventional cardiologist based on lesion characteristics, vessel anatomy, and operator preference.

### Brachytherapy procedure

Procedures were performed according to the vendor-recommended protocol. After successful balloon angioplasty of the restenotic lesion with either a plain balloon or a cutting/scoring balloon, beta radiation was delivered using a 7F guide catheter with the Beta-Cath 3.5F System (Novoste Corporation). The source train consisted of Strontium/Ytrium-90 seeds. To ensure complete lesion coverage and account for edge effects, the radiation source train length was selected to exceed the angioplasty segment by 10 mm on each end. Due to guide catheter placement issues or specific patient conditions, reduced margins were accepted in some cases.

Radiation dosing followed a vessel size-dependent protocol, with all doses prescribed at 2 mm from the radioactive source center: vessels with diameters ≤3.35 mm received 18.4 Gy, while vessel with diameters >3.35 mm received 23 Gy. Long lesions that required multiple contiguous dwells had an overlap segment of approximately 10 mm.

All lesion stenosis was visually estimated by the primary operator. The accepted threshold for residual stenosis postprocedure was <20%.

### Antithrombotic regimen

All patients received periprocedural anticoagulation with either intravenous heparin (70 IU/kg or 5000 IU) or bivalirudin (0.75 mg/kg bolus followed by 1.75 mg/kg/h infusion). Additional doses were administered as needed to maintain therapeutic anticoagulation. The postprocedural antithrombotic regimen included clopidogrel (75 mg/d after a 300-600 mg loading dose) for 12 months and lifelong aspirin (75-100 mg/d) as part of our institutional policy. Prasugrel or ticagrelor could be substituted for clopidogrel based on guidelines adopted at time of the procedure and clinical considerations.^8^, ^9^, ^10^ Preloading with any of these medications was permitted.

### Study end points

Baseline demographic characteristics data, cardiovascular risk factors, medication use, and procedural characteristics were collected from electronic medical records. Angiographic parameters, including lesion length and reference vessel diameter, were visually estimated by certified experienced interventional cardiologists. The primary end point was TLR at 1 year. Secondary end points included 1- and 3-year mortality and major adverse cardiovascular events (MACE), defined as a composite of cardiac death, target vessel myocardial infarction, or TLR, stent thrombosis, and bleeding events as defined by the Bleeding Academic Research Consortium criteria.^11^

### Statistical analysis

Continuous variables are expressed as mean ± SD, and categorical variables are presented as frequencies and percentages. Comparisons between the plain balloon and cutting/scoring balloon groups were performed using *t* tests for continuous variables and χ^2^ or Fisher exact test for categorical variables. Standardized mean differences were calculated to assess the magnitude of differences between groups, with values >0.2 considered clinically meaningful. Time-to-event analyses were conducted using Kaplan-Meier methods, with differences between groups assessed by the log-rank test. Cox proportional hazards models were used to identify independent predictors of adverse events, adjusting for potential confounders. A 2-sided *P* value < .05 was considered statistically significant. All statistical analyses were performed using Python 3.12.7.

### Study oversight

The study protocol was approved by the Institutional Review Board of Houston Methodist Hospital (PRO00025173), and written informed consent was waived for all patients because it was a retrospective chart review study. The study was conducted in accordance with the Declaration of Helsinki and Good Clinical Practice guidelines.

## Results

### Baseline patient characteristics

Our analysis included a total of 219 patients, with 140 (63.9%) in the plain balloon group and 79 (36.1%) in the cutting/scoring balloon group. Patients in the cutting/scoring balloon group were older than those in the plain balloon group (67.0 ± 11.0 vs 64.0 ± 10.3 years; *P* = .047). There was a trend toward a lower proportion of men in the cutting/scoring balloon group (64.6% vs 75.0%; *P* = .13). Body surface area was comparable between groups (2.00 ± 0.24 vs 2.03 ± 0.33 m^2^; *P* = .36).

The prevalence of hypertension (96.2% vs 97.1%; *P* > .99) and hyperlipidemia (91.1% vs 93.6%; *P* = .69) was similar between the cutting/scoring balloon and plain balloon groups, respectively. The proportion of patients with diabetes mellitus was numerically lower in the cutting/scoring balloon group (58.2% vs 66.4%; *P* = .28), while the proportion with chronic kidney disease (CKD) was higher (27.8% vs 20.0%; *P* = .24). There was a trend toward lower prevalence of reduced left ventricular ejection fraction (<50%) in the cutting/scoring balloon group (15.8% vs 24.8%; *P* = .240), while the prevalence of chronic obstructive pulmonary disease was similar (8.9% vs 7.9%; *P* = .99). Active smoking rates were comparable (32.4% vs 31.6%; *P* > .99) (Table 1).

**Table 1:** Baseline characteristics.

Characteristic	Plain balloon (n = 140)	Cutting balloon (n = 79)	*P*
Age, y	64.03 ± 10.35	67.06 ± 11.01	.0471
Male sex	105 (75.0%)	51 (64.6%)	.1378
Body surface area, m^2^	2.03 ± 0.33	2.00 ± 0.24	.3615
EF <50 (reference: EF ≥50)	32 (24.8%)	9 (15.8%)	.2397
COPD	11 (7.9%)	7 (8.9%)	.9972
Hypertension	136 (97.1%)	76 (96.2%)	>.99
Hyperlipidemia	131 (93.6%)	72 (91.1%)	.6937
Diabetes	93 (66.4%)	46 (58.2%)	.2872
Chronic kidney disease	28 (20.0%)	22 (27.8%)	.2456
Current smokers	45 (32.4%)	25 (31.6%)	>.99

Values are mean ± SD or n (%).

COPD, chronic obstructive pulmonary disease; EF, ejection fraction.

### Procedural characteristics

Procedural characteristics were generally similar between the cutting/scoring balloon and plain balloon groups. The mean procedure duration was comparable (91.3 ± 41.8 vs 87.0 ± 38.7 minutes; *P* = .47), as was the contrast volume used (151 ± 72 vs 142 ± 55 mL; *P* = .36). The fluoroscopy radiation dose area product was similar in the 2 groups (13,453 ± 9465 vs 15,647 ± 10925 mGy/cm^2^; *P* = .15).

Some differences were observed in lesion characteristics. The treated vessel size was similar between groups (3.5 ± 0.5 vs 3.5 ± 0.7 mm; *P* = .438), as well as the lesion length (28.01 ± 18.62 vs 26.49 ± 17.16 mm; *P* = .55). Small vessel disease, defined as reference vessel diameter <2.75 mm, was similar (6.3% vs 10.7%; *P* = .402).

The left anterior descending artery was the most commonly treated vessel in both groups, with similar proportions (31.6% vs 30.7%; *P* > .99). The prevalence of left circumflex artery and right coronary artery treatment were comparable (31.6% vs 22.9%; *P* = .21 and 32.1% vs 19.0%; *P* = .053, respectively). Treatment of the left main coronary artery (10.1% vs 4.3%; *P* = .16) and treatment of the ramus intermedius were similar between groups (3.8% vs 3.6%; *P* > .99) (Table 2).

**Table 2:** Procedural characteristics.

Characteristic	Plain balloon (n = 140)	Cutting balloon (n = 79)	*P*
Procedure length, min	87.07 ± 38.77	91.37 ± 41.82	.4713
Radiation dose, mGy/cm^2^	15,647.0 ± 10,925.5	13,453.4 ± 9465.3	.1485
Contrast volume, mL	142.21 ± 55.71	151.28 ± 72.82	.3689
Vessel size, mm	3.49 ± 0.66	3.56 ± 0.54	.4375
Lesion length, mm	28.01 ± 18.62	26.49 ± 17.16	.5537
Left anterior descending artery	43 (30.7%)	25 (31.6%)	>.99
Left circumflex artery	32 (22.9%)	25 (31.6%)	.2066
Right coronary artery	45 (32.1%)	15 (19.0%)	.0526
Ramus intermedius	5 (3.6%)	3 (3.8%)	>.99
Left main coronary artery	6 (4.3%)	8 (10.1%)	.1588
Vessel size <2.75 mm	15 (10.7%)	5 (6.3%)	.4023

Values are mean ± SD or n (%).

### Clinical outcomes

At 1-year follow-up, several differences in clinical outcomes were observed between the cutting/scoring balloon and plain balloon groups, although most did not reach statistical significance. The primary end point of TLR occurred in 26.6% of patients in the cutting/scoring balloon group compared to 17.9% in the plain balloon group (*P* = .18). (Central Illustration)

**Central Illustration:**
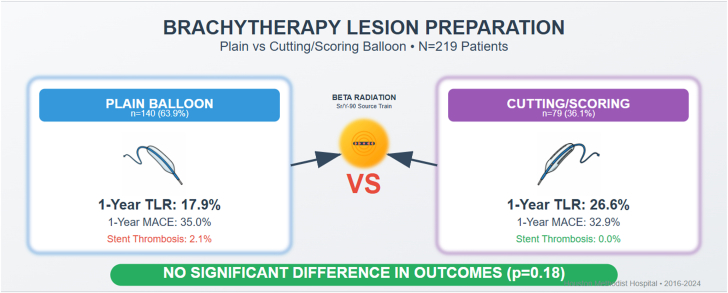
**Comparison of clinical outcomes between plain balloon and cutting/scoring balloon angioplasty for brachytherapy lesion preparation.** MACE, major adverse cardiac events; TLR, target lesion revascularization.

Although rare, stent thrombosis occurred exclusively in the plain balloon group (2.1% vs 0.0%; *P* = .48), as did bleeding complications (1.4% vs 0.0%; *P* = .74). Cardiac hospitalization rates were similar between groups (26.6% vs 27.1%; *P* > .99).

Myocardial infarction rates were nearly identical between the cutting/scoring balloon and plain balloon groups (8.9% vs 8.6%, *P* > .99). Cardiac death was infrequent in each group (1.3% vs 1.4%; *P* > .99). All-cause mortality at 1 year was similar in the cutting/scoring balloon group (3.8% vs 5.7%; *P* = 0.76). The composite end point of MACE showed comparable rates between groups (32.9% vs 35.0%; *P* = 0.87) (Table 3).

**Table 3:** One-year outcomes.

Outcome	Plain balloon (n = 140)	Cutting balloon (n = 79)	*P*
Target lesion revascularization	25 (17.9%)	21 (26.6%)	.1772
Thrombosis	3 (2.1%)	0 (0.0%)	.4809
Bleeding	2 (1.4%)	0 (0.0%)	.7432
Cardiac hospitalization	38 (27.1%)	21 (26.6%)	>.99
Myocardial infarction	12 (8.6%)	7 (8.9%)	>.99
All-cause mortality	8 (5.7%)	3 (3.8%)	.763
Cardiac death	2 (1.4%)	1 (1.3%)	>.99
MACE	49 (35.0%)	26 (32.9%)	.8693

Values are n (%).

MACE, major adverse cardiovascular event.

**Figure 1:**
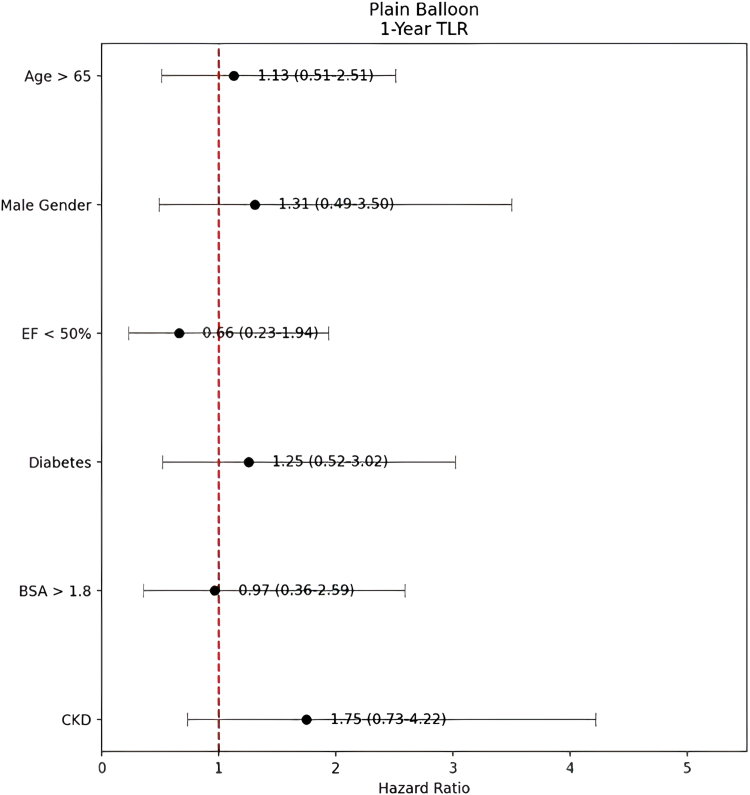
**Risk factors for TLR at 1 year following plain balloon angioplasty.** Forest plot showing hazard ratios (HR) and 95% confidence intervals for clinical predictors of 1-year TLR. The vertical dashed line represents HR = 1.0 (no effect). BSA, body surface area; CKD, chronic kidney disease; EF, ejection fraction; TLR, target lesion revascularization.

**Figure 2:**
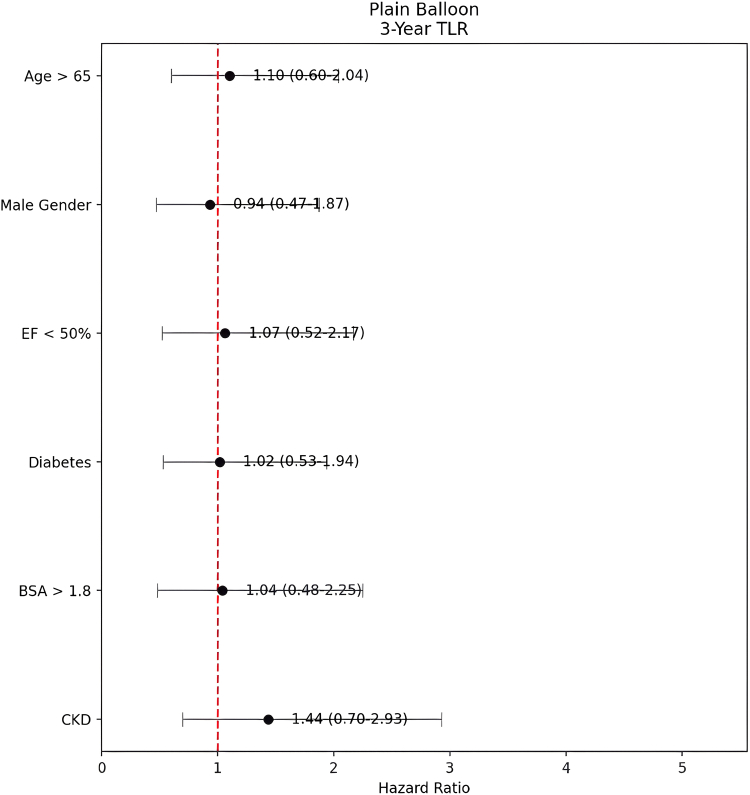
**Risk factors for TLR at 3 years after plain balloon angioplasty.** Forest plot displaying hazard ratios (HR) and 95% confidence intervals for clinical predictors of 3-year TLR. The vertical dashed line represents HR = 1.0 (no effect). BSA, body surface area; CKD, chronic kidney disease; EF, ejection fraction; TLR, target lesion revascularization.

In multivariable analysis, none of the examined variables demonstrated a statistically significant association with TLR at the 1-year and 3-year follow-ups. CKD, advanced age (>65 years), male sex, reduced ejection fraction (<50%), diabetes, and body surface area >1.8 were not independently associated with TLR risk in either group (Tables 4 and 5; Figures 1 and 2).

**Table 4:** One-year target lesion revascularization hazard ratio model for plain balloon.

Characteristic	HR	CI	*P*
Age >65 y	1.129426	0.51-2.51	.766
Male	1.307368	0.49-3.50	.594
Ejection fraction <50%	0.661534	0.23-1.94	.451
Diabetes	1.253755	0.52-3.02	.615
Body surface area >1.8 m^2^	0.966939	0.36-2.59	.947
Chronic kidney disease	1.75082	0.73-4.22	.213

HR, hazard ratio.

**Table 5:** Three-year target lesion revascularization hazard ratio model for plain balloon.

Characteristic	HR	CI	*P*
Age >65 y	1.103373	0.60-2.04	.753
Male	0.935419	0.47-1.87	.850
Ejection fraction <50%	1.065022	0.52-2.17	.862
Diabetes	1.017084	0.53-1.94	.959
Body surface area >1.8 m^2^	1.040913	0.48-2.25	.919
Chronic kidney disease	1.435531	0.70-2.93	.320

HR, hazard ratio.

**Figure 3:**
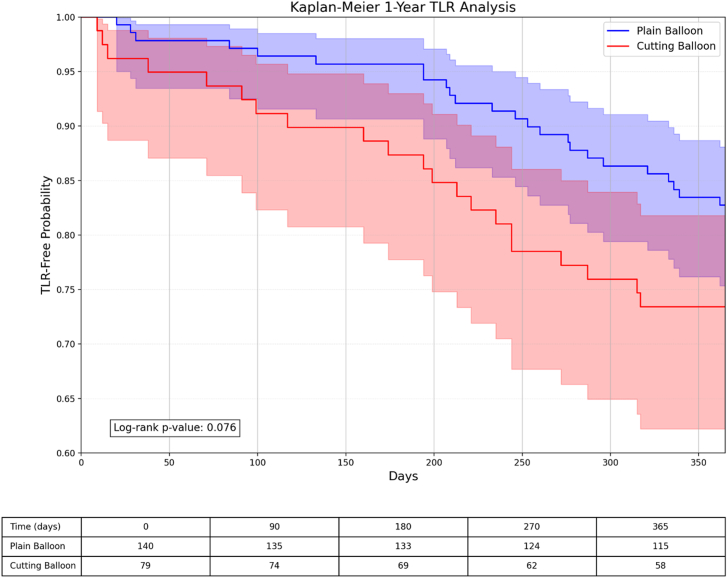
**Kaplan-Meier survival analysis comparing TLR-free probability at 1 year between plain balloon and cutting balloon angioplasty.** The risk table below shows the number of patients at risk at specified time intervals. TLR, target lesion revascularization.

**Figure 4:**
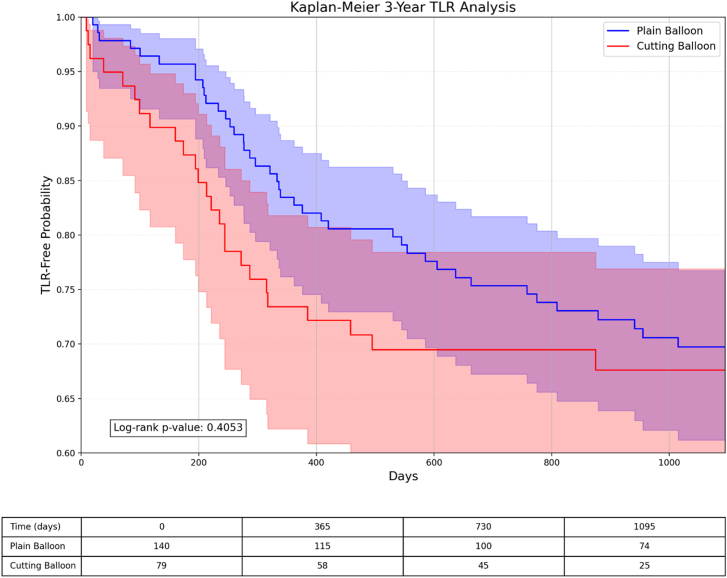
**Kaplan-Meier survival analysis comparing TLR-free probability at 3 years between plain balloon and cutting balloon angioplasty.** The risk table below shows the number of patients at risk at specified time intervals. TLR, target lesion revascularization.

**Figure 5:**
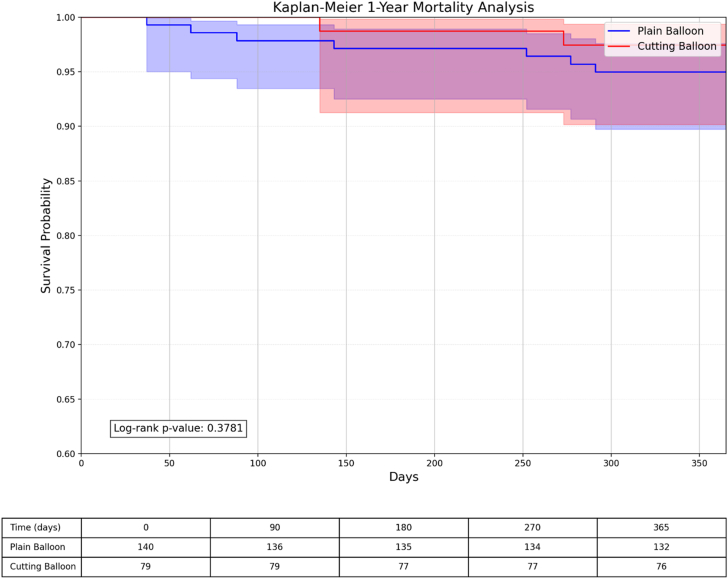
**Kaplan-Meier survival analysis comparing 1-year mortality between plain balloon and cutting balloon angioplasty.** The risk table below shows the number of patients at risk at specified time intervals.

**Figure 6:**
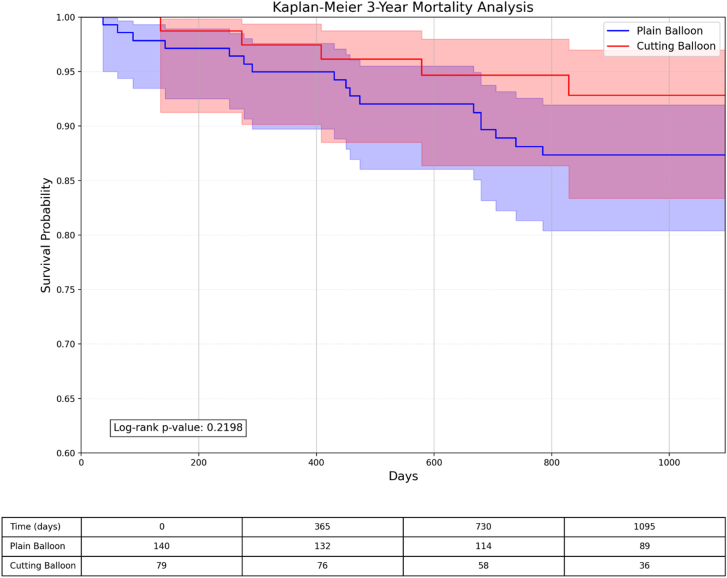
**Kaplan-Meier survival analysis comparing 3-year mortality between plain balloon and cutting balloon angioplasty.** The risk table below shows the number of patients at risk at specified time intervals.

Kaplan-Meier survival analyses demonstrated similar event-free survival patterns between the cutting/scoring balloon and plain balloon groups for TLR and all-cause mortality, with curves that remained parallel throughout the 1-year and 3-year follow-up periods (Figure 3, Figure 4, Figure 5, Figure 6).

## Discussion

Our analysis of the Houston Methodist Hospital Brachytherapy Registry, representing one of the largest single-center experiences of vascular brachytherapy for ISR, reveals interesting insights into the impact of lesion preparation strategy on clinical outcomes. Contrary to theoretical expectations, we found no significant advantage of cutting/scoring balloon angioplasty over conventional plain balloon angioplasty for lesion preparation before brachytherapy, and in fact observed a numerically higher, though not statistically significant, rate of TLR in the cutting/scoring balloon group. Our results align with previous studies demonstrating comparable outcomes between modified balloon techniques and conventional approaches across various lesion types, with no statistically significant differences observed.^12^

The higher numerical rate of 1-year TLR in the cutting/scoring balloon group (26.6% vs 17.9%) was unexpected given the theoretical advantages of these specialized balloon technologies, although it disappeared at 3-year follow-up. A possible explanation is that operators prefer to use these specialized balloons for longer, highly resistant lesions that may inherently carry a higher risk of recurrence. This selection bias could have influenced the observed outcomes independent of the balloon type itself.

Patient characteristics differed somewhat between groups, with older patients in the cutting/scoring balloon group (67.1 ± 11.0 vs 64.0 ± 10.4 years; *P* = .047), but clinically this difference may be irrelevant. There was a also numerically higher prevalence of CKD in the cutting/scoring balloon group (27.8% vs 20.0%; *P* = .246). Both advanced age and renal dysfunction have been associated with poorer outcomes after coronary interventions and may potentially contribute to the observed differences in TLR rates.^10^^,^^13^

Technically challenging lesion subsets, including left main coronary artery involvement and small vessel disease, were similarly distributed across both the conventional balloon and cutting/scoring balloon cohorts. Long lesions (>20 mm) were also comparably represented between groups, with mean lesion lengths of 28.01 ± 18.62 mm for plain balloons versus 26.49 ± 17.16 mm for cutting balloons (*P* = .55), reflecting the complex nature of the treated population. This balanced distribution of high-risk anatomical features suggests that differences in outcomes between preparation strategies may not be attributed to disparities in lesion complexity, strengthening the validity of our comparative analysis.

The mechanism of action of cutting/scoring balloons, while theoretically advantageous for plaque modification, may have complex interactions with the subsequent radiation therapy. The controlled incisions or scoring created in the vessel wall might alter the tissue response to radiation, potentially affecting the antiproliferative efficacy of brachytherapy. This hypothesis is speculative but merits investigation in future mechanistic studies.

Despite these considerations, it is important to note that the observed difference in TLR rates did not reach statistical significance, and the overall MACE rates were remarkably similar between groups (32.9% vs 35.0%; *P* = .87). This suggests that while specific components of adverse outcomes may differ, the overall clinical benefit of brachytherapy is largely independent of the balloon type used for lesion preparation.

The safety findings in our study are also noteworthy. The exclusive occurrence of stent thrombosis (2.1% vs 0.0%) and bleeding complications (1.4% vs 0.0%) in the plain balloon group, although not statistically significant given the low event rates, suggests a potential safety advantage of cutting/scoring balloons. These specialized balloons may create more controlled plaque modification with fewer dissections requiring additional stenting, potentially reducing the risk of late thrombotic events.^14^^,^^15^ The difference can be lesion-related as well.

These findings have important implications for clinical practice. While cutting/scoring balloons offer theoretical advantages and are widely used for resistant lesions, our results do not support their routine use for lesion preparation before brachytherapy based solely on expected improvements in clinical outcomes. Instead, balloon selection should be individualized based on lesion characteristics, vessel anatomy, and operator experience. The similar overall event rates between groups suggest that both approaches can achieve acceptable results when used appropriately.

The comparable outcomes observed with both balloon types may also have economic implications. Cutting and scoring balloons are significantly more expensive than conventional balloons, and their use adds to procedural costs. In the absence of clear clinical benefits, judicious use of these specialized devices may help optimize resource utilization without compromising patient outcomes.

Future research directions emerging from our findings include investigating whether specific lesion subsets might particularly benefit from cutting/scoring balloon preparation before brachytherapy, exploring the tissue-level effects of different balloon types on subsequent radiation response, and determining whether longer-term outcomes beyond 1 year might reveal more pronounced differences between preparation strategies.

### Limitations

Our study has several important limitations that should be acknowledged. First, as a single-center retrospective analysis, our findings may reflect institutional expertise and practice patterns specific to our tertiary care center, potentially limiting generalizability to other clinical settings. The nonrandomized nature of balloon selection introduces the possibility of selection bias, despite our efforts to control for known confounders through multivariate analysis.

Second, the relatively small sample size, particularly in the cutting/scoring balloon group (n = 79), may have limited our ability to detect more subtle differences in outcomes or to identify additional independent predictors of adverse events. This is particularly relevant for rare events such as stent thrombosis and bleeding complications, for which the observed differences did not reach statistical significance.

Third, our analysis did not distinguish between cutting balloons and scoring balloons, which have different mechanical properties and may have distinct effects on vessel wall preparation. Further studies with larger sample sizes would be needed to determine whether these specific balloon subtypes have differential impacts on outcomes.

Fourth, the heterogeneity of the coronary lesion characteristics and prior interventional history in our study population may have influenced outcomes independent of the balloon type used for preparation, although recent evidence suggests that multiple stent layers are not predictive for 1-year TLR.^16^^,^^17^ Although we attempted to account for measurable factors, unmeasured variables related to lesion complexity and vascular biology may have confounded our results.

Fifth, our follow-up period of 1 year, while providing important insights into medium-term outcomes, may not capture later adverse events that could influence the overall assessment of different preparation strategies. Longer-term follow-up would be necessary to determine the durability of treatment effects and to identify any late differential outcomes.

Sixth, we were not able to verify prior brachytherapy for all patients, as procedures performed before 2016 were not digitally archived and thus inaccessible.

Finally, our study did not include comprehensive data on procedural complications immediately after balloon angioplasty, such as dissections or the need for bailout stenting, which might have provided additional insights into the acute efficacy and safety of different balloon types.

## Conclusion

Our analysis demonstrates that patients undergoing vascular brachytherapy for ISR experience similar overall clinical outcomes at 1 year regardless of whether lesion preparation is performed with conventional plain balloon angioplasty or specialized cutting/scoring balloon angioplasty. Although the cutting/scoring balloon group showed a numerically higher rate of TLR, this difference did not reach statistical significance, and MACE rates were nearly identical between groups.

The exclusive occurrence of stent thrombosis and bleeding complications in the plain balloon group, though not statistically significant given the low event rates, suggests a potential safety advantage of cutting/scoring balloons that warrants further investigation in larger studies.

These findings suggest that balloon selection for lesion preparation before brachytherapy should be individualized based on specific lesion characteristics and operator experience rather than routinely favoring either approach. The similar overall outcomes with both strategies provide interventional cardiologists with flexibility in their approach to these challenging cases.

Future research should focus on identifying specific lesions or patient subsets that might particularly benefit from specialized balloon technologies, exploring the tissue-level effects of different preparation strategies on radiation response, and determining whether longer-term outcomes might reveal more pronounced differences between approaches. Such investigations could help refine treatment algorithms and optimize outcomes for patients with recalcitrant ISR requiring brachytherapy.

## Declaration of competing interest

The authors declared no potential conflicts of interest with respect to the research, authorship, and/or publication of this article.
